# Upregulation of *GPR133* expression impaired the phagocytosis of macrophages in recurrent spontaneous miscarriage

**DOI:** 10.1080/15592294.2024.2337087

**Published:** 2024-04-02

**Authors:** Jia-Xue Sun, Yongli Yao, Wen-Xuan Li, Xin Su, Haoyu Yang, Zhouping Lu, Chenfei Liu, Xiang-Hong Xu, Liping Jin

**Affiliations:** Department of Biobank, Shanghai Key Laboratory of Maternal Fetal Medicine, Shanghai Institute of Maternal-Fetal Medicine and Gynecologic Oncology, Shanghai First Maternity and Infant Hospital, School of Medicine, Tongji University, Shanghai, P.R, China

**Keywords:** Recurrent spontaneous miscarriage, decidual macrophage, GPR133, methylation, phagocytosis

## Abstract

Decidual macrophages are the second-largest immune cell group at the maternal-foetal interface. They participate in apoptotic cell removal, and protect the foetus from microorganisms or pathogens. Dysfunction of decidual macrophages gives rise to pregnancy complications such as preeclampsia and recurrent spontaneous miscarriage (RSM). However, the mechanisms by which decidual macrophages are involved in the occurrence of adverse pregnancy outcomes have not been elucidated. Here we integrated DNA methylation and gene expression data from decidua macrophages to identify potential risk factors related to RSM. *GPR133* was significantly hypomethylated and upregulated in decidual macrophages from RSM patients. Further demethylation analysis demonstrated that *GPR133* expression in decidual macrophages was significantly increased by 5-Aza-dC treatment. In addition, the influence of GPR133 on the phagocytic ability of macrophages was explored. Phagocytosis was impaired in the decidual macrophages of RSM patients with increased *GPR133* expression. Increased *GPR133* expression induced by demethylation treatment in the decidual macrophages of healthy control patients led to a significant decrease in phagocytic function. Importantly, knockdown of *GPR133* resulted in a significant improvement in the phagocytic function of THP-1 macrophages. In conclusion, the existing studies have shown the influence of *GPR133* on the phagocytic function of decidual macrophages and pregnancy outcomes, providing new data and ideas for future research on the role of decidual macrophages in RSM.

## Introduction

Recurrent spontaneous miscarriage (RSM) refers to two or more consecutive spontaneous abortions with the same spouse before the 20th week of gestation [1]. RSM accounts for approximately 2–5% of the total pregnancies [2]. Although concentrated research has identified numerous causes of RSM, such as chromosomal abnormalities, abnormal reproductive structure, endocrinopathies, and thrombophilic disorders, 50% of RSM cases are still unexplained [3]. Exploring the pathogenesis of unexplained RSM and providing early intervention are critical for ensuring a better pregnancy outcome.

The immune tolerogenic microenvironment that is formed at the maternal-foetal interface has an irreplaceable effect on the maintenance of pregnancy and the avoidance of foetal rejection. Various immune cells, such as natural killer (NK) cells, macrophages, and T cells, are involved in immune-tolerance to ensure successful pregnancies. Macrophages are the second-largest immune cell group and comprise 20–30% of all immune cells at the maternal-foetal interface [4]. Macrophages are located close to invading trophoblast cells and remodel the uterine spiral artery [5]. They participate in embryo implantation, trophoblast invasion, uterine spiral artery remodelling, apoptotic cell removal, and protection of the foetus from microorganisms or pathogens [6–8].

With the development of epigenetics, studies of DNA methylation in the uterine immune tolerogenic microenvironment have provided a new perspective on the pathogenesis of RSM. A genome-wide DNA methylation analysis was performed on the placental villi of the RSM, and many significantly differentially methylated regions were found near dysregulated genes that were enriched in the immune response pathway [9]. A comparison of blood monocytes and decidual macrophages at the maternal-foetal interface revealed that the genes encoding markers of classical macrophage activation were hypermethylated, while those encoding alternative macrophage activation markers were hypomethylated in the decidual macrophages [10], indicating that the proper function of decidual macrophages is partly associated with their DNA methylation pattern. However, research on the DNA methylation of decidual macrophages of RSM is still lacking.

We hypothesized that distinct changes in DNA methylation occur in decidual macrophages between RSM patients and healthy controls and that differential DNA methylation patterns will provide clues for further understanding the immunological characteristics of decidual macrophages during human pregnancy. In the present study, we compared the DNA methylome of decidual macrophages from RSM patients and healthy control subjects. Subsequently, the differentially expressed genes in decidual macrophages was determined. After the integration of methylomes and transcriptomes, five genes (*EPAS1*, *GPR133*, *RHBDF1*, *SLC44A4*, and *TRPC4*) were identified and verified via qRT – PCR. Moreover, a bisulphate-sequencing PCR (BSP) assay was performed, and reduced methylation levels of the *GPR133* gene were observed in the RSM patients compared to those in the healthy controls. In addition, the influence of *GPR133* on the phagocytic ability of macrophages was explored. Collectively, these findings illustrated that the abnormal methylation and expression levels of *GPR133* may be related to RSM.

## Materials and methods

### Sample collection

First-trimester human decidual tissues were collected from clinically healthy pregnant women and pregnant women who were admitted to the Shanghai First Maternity and Infant Hospital for unexplained RSM. The unexplained RSM group comprised 21 women with a history of two to four spontaneous miscarriages during early pregnancy (6–11 weeks gestation). The mean age of the patients was 32.14 ± 0.68 years. Patients were diagnosed with unexplained RSM after any verifiable cause, including chromosomal abnormalities, anatomic deformation, infection, endocrine or metabolic diseases, or autoimmune response, were excluded. The control group comprised 21 randomly selected women who underwent legal termination for nonmedical reasons (6–11 weeks gestation) at the same facility during the same period. The mean age of the individuals in the control group was 30.04 ± 1.17 years. All the control subjects had one or two living children and no history of spontaneous miscarriage, ectopic pregnancy, preterm delivery or stillbirth. In all the patients, foetal heart activity was verified, and the embryonic karyotype was normal. There was no significant difference in age or gestation time between the unexplained RSM group and the control group. Written informed consent was obtained from all patients before sampling. The patient demographics are summarized in Supplementary Table S1. The study protocol was approved by the Scientific and Ethics Committee of the Shanghai First Maternity and Infant Hospital affiliated with Tongji University. All decidual samples for this study were collected after curettage and transported to the laboratory under sterile conditions within 1 hour.

### Enrichment of primary decidual macrophages

Decidual mononuclear cells were obtained from the decidual tissues digested with collagenase type IV (1.0 mg/ml, Sigma, #C5138-1 G) and DNase I (150 U/ml, Sigma, #DN25-1 G) for 40 min at 37°C followed by discontinuous Percoll gradient centrifugation (GE, 17-5445-01). Then, the decidual mononuclear cells were placed in culture flasks at 37°C with 5% CO_2_ for 30 min to remove the adherent decidual stromal cells. The macrophages were enriched from the isolated decidual mononuclear cells using anti-CD14 reactive magnetic beads (Miltenyi Biotec, #130-050-201). The purity of the enriched macrophages was analysed by staining the cells with monoclonal antibodies specific for CD45 (BioLegend, #304012), CD14 (BioLegend, #325604) and CD68 (BioLegend #137010) for 30 min at 4°C.

### DNA methylation analysis

Genomic DNA was isolated from decidual macrophages, converted by sodium bisulphate modification and hybridized to Illumina Human Methylation 850k arrays following the manufacturer’s protocols at Shanghai OE Biotech Co., Ltd. (Shanghai, China).

### Differential methylation analysis

The COHCAP package in R (https://sourceforge.net/projects/cohcap) was used to identify the differential methylation sites between diseased and healthy samples that are more likely to regulate downstream gene expression. |Diffscore|> 13 and |Δβ| > 0.17 were considered differentially methylated CpG sites. To determine the biological function of the differentially methylated genes (DMGs), we conducted Gene Ontology (GO) enrichment analysis via the online tool DAVID (https://david.ncifcrf.gov/tools.jsp). A false discovery rate (FDR) < 0.05 was set as the cut-off for selecting significantly enriched functional GO pathways.

### Transcriptomic analysis

Transcriptome profiles were obtained using the Agilent SurePrint G3 Human Gene Expression 8 × 60K microarray platform at Shanghai Biotechnology Corporation, China.

### Differential expressed gene analysis

Based on the gene expression microarray data, differentially expressed genes (DEGs) between the samples from women with RSM and healthy controls were identified. A fold change ≥ 2 and a *p* value < 0.05 were set as the thresholds for differential expression. A volcano plot was generated using Bioconductor (https://bioconductor.org/biocLite.R). The ‘pheatmap’ package in R statistical software was used to construct heatmaps.

### Correlation analysis between DMGs and DEGs

We identified genes that were not only differentially expressed but also differentially methylated to analyse the association between gene expression and DNA methylation. DNA methylation is negatively correlated with gene expression in many cases. In this study, we identified DEGs whose expression and DNA methylation were inversely regulated.

### Real-time quantitative polymerase chain reaction (qRT – PCR)

Total RNA was isolated using TRIzol reagent (Invitrogen, #15596018). First-strand cDNA was synthesized from 500 ng of total RNA with a PrimeScript™ II 1st Strand cDNA Synthesis Kit (Takara, #6210A). qRT – PCR was performed using a StepOne analyser (Applied Biosystems, Carlsbad, CA, USA) with TB Green™ Premix Ex Taq™ (Takara, #RR420A) according to the manufacturer’s protocol. *ACTB* was chosen as the reference gene. The qRT – PCR primers used are listed in Supplementary Table S2.

### DNA extraction, bisulphite treatment and promoter methylation analysis

Genomic DNA was extracted using an AxyPrep Multisource Genomic DNA MinPrep Kit according to the manufacturer’s protocol (Axygen Scientific, #AP-MN-MS-GDNA-250 G, Union City, CA, USA). Bisulphite treatment of each DNA sample was performed using a BisulFlash DNA Modification Kit (Epigentek Group Inc., #P-1026-050; Farmingdale, NY, USA). CpGenome Universal Methylated DNA (Millipore, #S7820; Billerica, MA, USA) and synthetic DNA fragments were used as fully methylated and unmethylated controls, respectively. The BSP method [11] was used to analyse the methylation levels of the promoters of five genes (*EPAS1*, *GPR133*, *RHBDF1*, *SLC44A4*, and *TRPC4*). PCR was carried out with the following program: a 5 min cycle at 95°C; 30 cycles of 30 s at 95°C, 30 s at 59°C, and 30 s at 72°C; and a final extension cycle of 7 min at 72°C. PCR products were purified using a gel extraction kit. The resulting products were subsequently cloned and sequenced. The DNA methylation rate of a given clone sequence was calculated as the proportion of methylated CpG sites to the total number of CpG sites in the sequence. The BSP primers used are listed in Supplementary Table S2.

### Immunofluorescence microscopy

Decidual macrophages were seeded in glass bottom cell culture dishes. Then, the cells were fixed with 4% paraformaldehyde in PBS for 20 min and permeabilized with 0.2% Triton X-100 in PBS for 10 min at room temperature. Following washing in PBS and blocking by incubation in normal goat serum, the cells were incubated overnight with mouse anti-CD68 (Abcam, #ab955; 1:150; Cambridge, MA, USA) and rabbit anti-GPR133 (Abbkine, #ABP54583; 1:500; Wuhan, China) antibodies simultaneously. Following washing, the cells were incubated for 1 h with Alexa Fluor 488-conjugated goat anti-rabbit secondary antibodies (Invitrogen, #A11070, 1:1000, Carlsbad, CA, USA) and Alexa Fluor Plus 555-conjugated goat anti-mouse secondary antibodies (Invitrogen, #A32727, 1:1000). Nuclei were stained with DAPI. Fluorescence images were obtained using a confocal microscope (TCS SP8; Leica, Wetzlar, Germany). Quantification of the fluorescence signal for GPR133 was performed using ImageJ. The mean integrated density of each field was normalized to the number of CD68-positive cells in the field.

### 5-aza-dC treatment

Decidual macrophages were seeded in cell culture dishes and subjected to continuous demethylation treatment with 5-Aza-dC (Sigma, #189825). The 5-Aza-dC-containing medium was replaced every 24 h. After 72 h of treatment with 1.5 μM, 5 μM or 20 μM 5-Aza-dC, the decidual macrophages were harvested for RNA extraction or phagocytosis. The control cells were incubated without 5-Aza-dC.

### Cell culture

THP-1 cells were purchased from the Cell Bank of the Chinese Academy of Sciences and cultured in RPMI-1640 medium supplemented with 10% FBS (Gibco, Life Technologies, Grand Island, NY, USA) and 1% penicillin/streptomycin at 37°C in 5% CO_2_ humidified air. The HTR-8/SVneo cell line used in this study was a kind gift from Dr. C.H. Graham at Queen’s University, Canada [12]. HTR-8/SVneo cells were cultured in Dulbecco’s modified Eagle Medium supplemented with nutrient mixture F-12 (Gibco), 10% FBS and 1% penicillin/streptomycin at 37°C in humidified air containing 5% CO_2_.

### Small interfering RNA (siRNA) transfection

siRNA (Genepharma, Shanghai, China) was used to investigate the effects of *GPR133* on the phagocytic functions of THP-1 cells. The sequences of the siRNAs used were as follows: siGPR133 sense: 5’-AGUCACGCUUCUCUAUUACTT-3;’ negative control (NC) sense: 5’-UUCUCCGAACGUGUCACGUTT-3.’ Prior to transfection, M1-like macrophages were induced from THP-1 cells by treatment with LPS (100 ng/ml; Absin, #abs47014848-5 mg) and IFN-γ (20 ng/ml; R&D Systems, #285-GMP-100) for 18 hours after phorbol 12-myristate 13-acetate (100 nM; Sigma, #P8139) treatment for 24 hours. THP-1 macrophages were transfected with 50 nmol/L siRNA using Lipofectamine 3000 transfection reagent (Invitrogen, #L3000015) in Opti-MEM I reduced serum medium according to the manufacturer’s instructions. Nontargeting siRNA (50 nmol/L) was used as the NC.

### Phagocytosis of apoptotic cells

An apoptotic cell model was constructed in HTR-8/SVneo cells treated with H_2_O_2_ (800 μM; Sigma, #STBH9407) for 8 hours. The apoptosis-induced HTR-8/SVneo cells were then labelled with CellTracker™ Orange CMRA probe (1:10000; Life Technology, #C34551; NY, USA) for 30 mins and subsequently incubated with decidual macrophages or THP-1 macrophages, at a ratio of 1:1 for 120 minutes at 37°C. Macrophages and HTR-8/SVneo cells were collected and then stained with fluorochrome-conjugated antibodies against macrophages for 30 minutes at 4°C according to the recommended dosage. Following incubation, the cells were washed in PBS. The flow cytometry data were collected immediately by a BD flow cytometer and then analysed with FlowJo version 10.8 software. The efficiency of phagocytosis was calculated as follows: (CMRA^+^ macrophages/total macrophages) ×100%.

### Statistical analysis

The statistical differences between cases and controls were evaluated by Student’s t-test using GraphPad Prism version 6 (GraphPad Software, San Diego, CA, USA). A two- tailed *p* < 0.05 was considered to indicate statistically significant difference.

## Results

### Overall changes in genomic DNA methylation in decidual macrophages from patients with RSM

**Figure 1.**
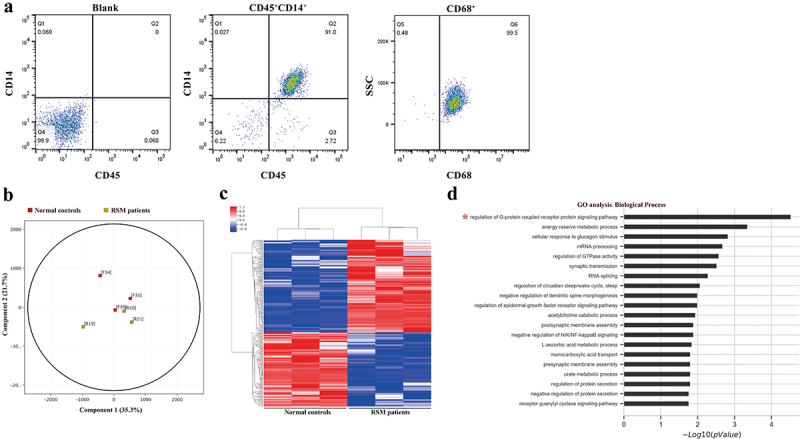
Differentially methylated CpG sites between healthy controls and RSM patients. (a) The purity of the isolated decidual macrophages was detected by FACS. The cells that were positive for CD45 and CD14 were considered decidual macrophages. CD45^+^CD14^+^ cells were also positive for CD68, a pan-macrophage marker. (b) Principal component analysis clearly showed separation between normal macrophages (red square, *n* = 3) and RSM macrophages (yellow square, *n* = 3). (c) Heatmap generated from clustering- analysis of microarray data illustrating differentially methylated DNA sites in the RSM patient group relative to the control group. Blue and red represent hypomethylation and hypermethylation, respectively, whereas white indicates no change in methylation relative to the control. Each row represents the beta value of a differentially methylated CpG site and each column represents one sample. (d) Pathways enriched in differentially methylated genes were determined using the GO database.

Decidual macrophages were sorted by CD14 microbeads and then stained with CD45, CD14 and CD68 antibodies to determine the purity. The results showed that 91% of the cells were CD45^+^CD14^+^ (Figure 1(a)). CD68, a pan-macrophage marker, was also used to characterize decidual macrophage populations. The percentage of CD68^+^ cells among all CD45^+^CD14^+^ cells was 99.5% (Figure 1(a)).

To assess differences in methylation in decidual macrophages between patients with RSM and control individuals, we conducted a genome-wide analysis of DNA methylation in samples from the two groups using the Illumina Human Methylation 850k BeadChip. Principal component analysis (PCA) clearly revealed differences in decidual macrophages between normal subjects and RSM patients (Figure 1(b)). Statistical analysis revealed a total of 279 loci with differential DNA methylation between the two groups (defined using |Diffscore| > 13 and |Δβ| > 0.17). Among these differential methylation sites, 156 sites were hypermethylated, and 123 sites were hypomethylated compared to the methylation status of those sites in the control subjects. A heatmap was generated from clustering-analysis illustrating differentially methylated DNA sites in the RSM patient group relative to the control group (Figure 1(c)). Pathways enriched in differentially methylated genes were determined using the GO database (http://geneontology.org/). A total of 33 differential methylation sites in the 5’UTR were found with available biological data corresponding to 29 genes. The corresponding biological functions of these genes were obtained from the Database for Annotation, Visualization, and Integrated Discovery database. Among the 105 biological process terms, the following 2 terms were significantly enriched in the differentially methylated genes (FDR-corrected *p* value < 0.05): GO:0008277, regulation of G-protein coupled receptor protein signalling pathway (FDR-corrected *p* value = 0.003) and GO:0006112, energy reserve metabolic process (FDR-corrected *p* value = 0.023) (Figure 1(d)).

### Overall changes in gene expression in decidual macrophages from patients with RSM

**Figure 2.**
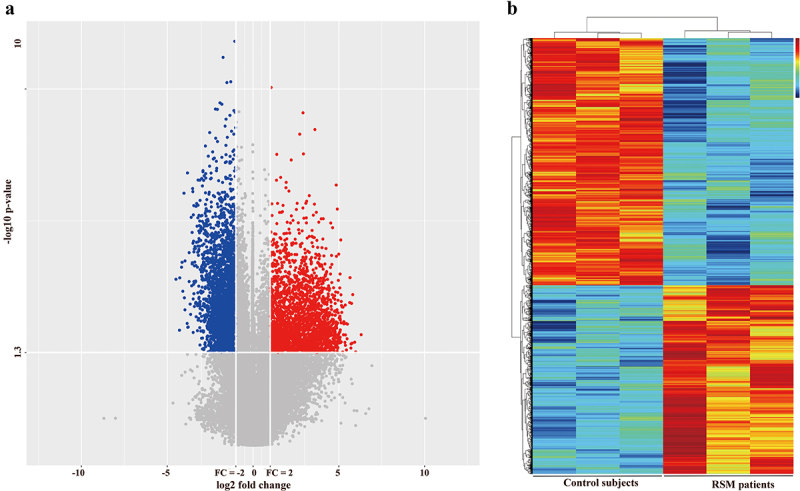
Differentially expressed genes between healthy controls and RSM patients. (a) Volcano plot showing that the two vertical lines are the 2-fold change boundaries and that the horizontal line is the statistically significant boundary (*p* < 0.05). Genes with a fold change ≥ 2 and statistical significance are marked with red and blue dots, respectively. (b) A heatmap was generated. Each line represents a single gene and each column represents a sample. Different colours indicate different expression levels. Red and black indicate upregulation and downregulation, respectively.

To assess differences in gene expression in decidual macrophages between RSM patients and control individuals, transcriptome profiles were obtained from the two groups of patients using the Agilent SurePrint G3 Human Gene Expression 8 × 60K microarray platform. DEGs were selected through a ≥ 2-fold change cut-off using a t test (*p* < 0.05). We detected 4267 differentially expressed genes, which included 1848 upregulated genes and 2419 downregulated genes. A volcano plot was constructed to show up- or downregulated genes (Figure 2(a)). A heatmap diagram was constructed to show the correlations between the genes (Figure 2(b)).

### Association analysis between differential methylation and DEGs

A Pearson correlation test was performed between the standard signal value of mRNA expression and the average beta value of methylation. The results showed that the expression levels of 13 genes were significantly negatively correlated with the methylation levels (Table 1). There were five genes (*EPAS1*, *GPR133*, *RHBDF1*, *SLC44A4*, and *TRPC4*) with significantly reduced methylation and eight genes with significantly increased methylation in RSM decidual macrophages. Due to the significantly reduced expression of DNA methyltransferase 1 (DNMT1) (Supplementary Table S3), which is involved in the regulation of DNA methylation, five genes (*EPAS1*, *GPR133*, *RHBDF1*, *SLC44A4*, and *TRPC4*) with significantly reduced methylation in RSM decidual macrophages were selected for subsequent validation.

**Table 1.** Genes that showed significantly negatively correlations between gene expression levels and methylation levels.

			Standard signal value of gene expression						Average beta value of methylation					
Genes	Correlation coefficients*	P value	NOR-1	NOR-2	NOR-3	RSM-1	RSM-2	RSM-3	NOR-1	NOR-2	NOR-3	RSM-1	RSM-2	RSM-3
CHD7	−0.9034	0.0135	7.7773	7.8307	7.6915	6.0616	6.8288	7.1281	0.1951	0.0870	0.0724	0.8743	0.8497	0.2455
CUL3	−0.9318	0.0068	10.2467	10.2636	10.3889	10.6490	10.8356	10.9306	0.9218	0.9302	0.9149	0.5209	0.5366	0.5364
EPAS1	−0.9219	0.0089	10.7561	11.7016	11.2800	13.7953	12.9335	13.2733	0.7747	0.7305	0.8375	0.5319	0.6738	0.5387
GPR133	−0.9184	0.0097	4.3136	4.7622	5.4180	10.0161	7.5025	6.4230	0.8213	0.8586	0.8382	0.0850	0.4123	0.3146
HLA-DRB5	−0.8577	0.0289	14.0453	14.3329	14.7608	11.4989	13.7533	12.1621	0.0885	0.1800	0.0551	0.5199	0.2851	0.2501
KANSL2	−0.8746	0.0226	5.9921	5.4751	5.6514	4.9061	5.2187	5.6195	0.0642	0.0823	0.0106	0.8595	0.3943	0.2275
OSBPL3	−0.8578	0.0289	11.1476	10.9941	11.3066	9.1003	9.2230	9.5195	0.4119	0.3076	0.3265	0.6316	0.4610	0.5238
RHBDF1	−0.8437	0.0348	3.3298	4.1243	3.7782	7.9113	6.4844	6.4800	0.5361	0.8785	0.5447	0.0672	0.0629	0.0448
SLC43A1	−0.9035	0.0135	5.9974	6.5672	6.4230	4.9705	5.3556	5.4928	0.2183	0.1369	0.0517	0.8928	0.3614	0.6089
SLC44A4	−0.8698	0.0243	0.8271	0.8936	2.2027	5.9111	5.6812	6.0643	0.7960	0.4244	0.8040	0.0924	0.0701	0.0563
TEKT5	−0.8165	0.0474	3.3634	3.1833	3.2596	2.5485	5.7920	5.4972	0.4635	0.8220	0.8023	0.4360	0.0351	0.0394
TRPC4	−0.8906	0.0173	2.2248	3.5713	2.6437	9.0140	5.8967	6.1391	0.7570	0.8052	0.7936	0.2199	0.2197	0.2707
TTTY14	−0.9613	0.0022	9.3318	8.9896	9.0478	7.8355	8.4155	8.4853	0.2170	0.3018	0.3532	0.6873	0.5698	0.4137

Note: *A Pearson correlation test was performed between the standard signal value of mRNA expression and the average beta value of methylation. Correlation coefficients are shown for each gene.

### Validation of the microarray results via qRT – PCR

**Figure 3.**
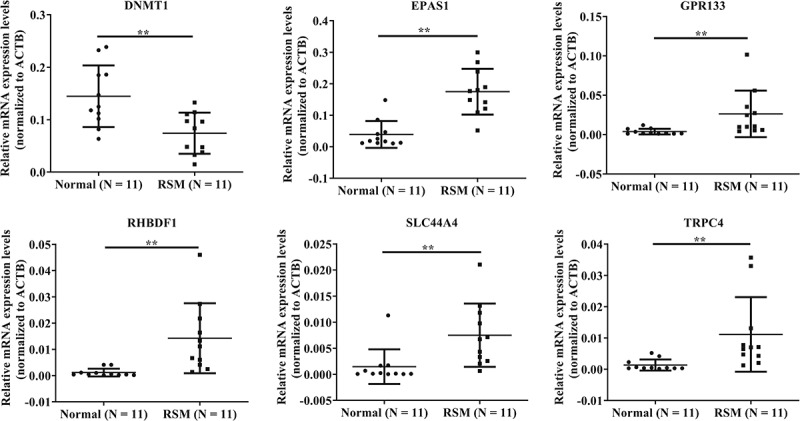
Validation of the microarray results via qRT – PCR. the data are presented as the means ± SDs and were analysed by two-sided unpaired Student’s t tests. **p < 0.01.

The microarray data for *DNMT1*, *EPAS1*, *GPR133*, *RHBDF1*, *SLC44A4*, and *TRPC4* were validated via qRT – PCR analysis. We observed that all six genes examined in the microarray were consistent with the RT-qPCR results. As shown in (Figure 3), *DNMT1* expression was significantly lower in the RSM decidual macrophages than in the control decidual macrophages. The expression of *EPAS1*, *GPR133*, *RHBDF1*, *SLC44A4*, and *TRPC4* in RSM decidual macrophages was significantly greater than that in normal controls (Figure 3).

### Validation by sulphite sequencing

**Figure 4.**
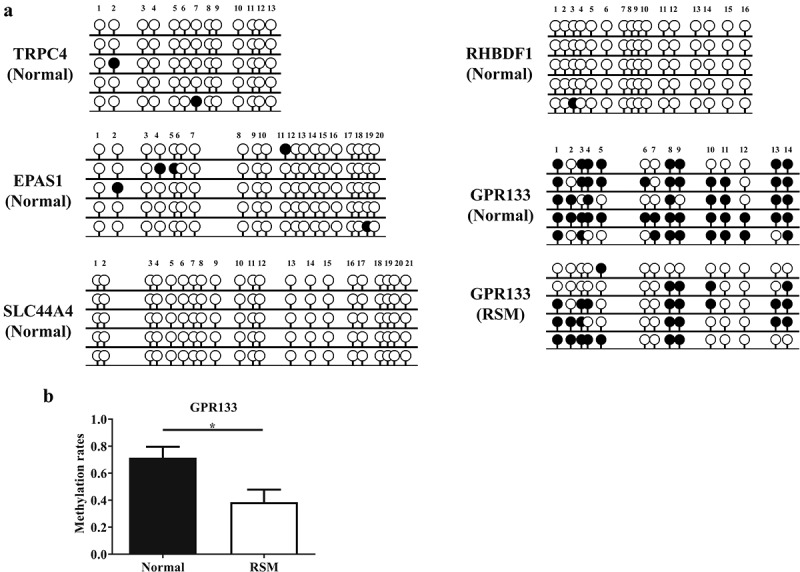
Methylation status was evaluated by BSP in decidual macrophages. (a) The methylation status of the CpG islands in five genes (*TRPC4*, *RHBDF1*, *EPAS1*, *SLC44A4* and *GPR133*) was evaluated via BSP in macrophages isolated from first-trimester human decidual tissues. (b) Methylation rates of the *GPR133* gene were assessed in decidual macrophages from normal individuals and RSM patients, and the statistical data are represented as the means ± SEMs of the sequenced clones. An unpaired Student’s t test was performed for significance testing. **p < 0.05.

Furthermore, the CpG dinucleotide-rich region in the promoter was analysed via BSP to determine the methylation status of five genes (*EPAS1*, *GPR133*, *RHBDF1*, *SLC44A4*, and *TRPC4*) in decidual macrophages. As shown in (Figure 4(a)), *GPR133* was hypermethylated, but the other four genes were hypomethylated in the decidual macrophages from healthy pregnant subjects. Moreover, reduced methylation levels of the *GPR133* gene were observed in RSM patients compared to normal controls (Figures 4(a,b)).

### GPR133 protein expression is increased in the decidual macrophages from RSM patients

**Figure 5.**
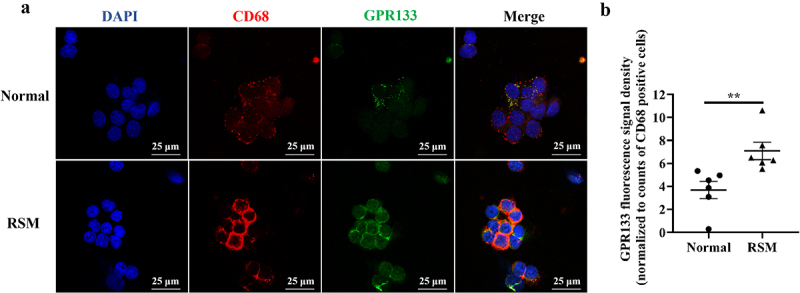
Increased expression of GPR133 was found in decidual macrophages from RSM patients. (a) The expression of GPR133 was examined in decidual macrophages from normal subjects (top row) and RSM patients (bottom row) using immunofluorescence microscopy. Scale bar = 25 μm. (b) Quantification of the fluorescence signal for GPR133 was performed using ImageJ. The mean integrated density of each field was normalized to the number of CD68-positive cells in the field. The statistical data are presented as the means ± SEMs and were analysed by two-sided unpaired Student’s t tests. **p < 0.01.

To detect the protein expression levels of GPR133 in decidual macrophages from healthy pregnant women and RSM patients, immunofluorescence staining was performed. The results showed that the expression of GPR133 in the decidual macrophages of RSM patients was greater than that in the decidual macrophages from healthy patients (Figure 5).

### Phagocytosis is reduced in the decidual macrophages of patients with RSM

**Figure 6.**
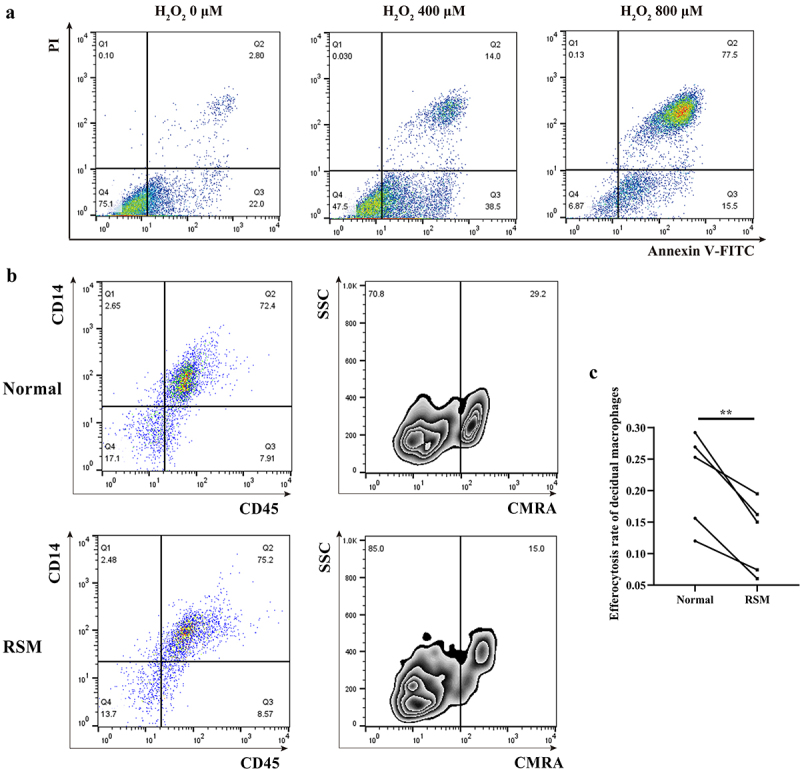
The percentage of apoptotic HTR-8/SVneo cells that underwent phagocytosis was estimated for first-trimester decidual macrophages from normal controls and RSM patients. (a) HTR-8/SVneo cells were seeded in 6-well culture plates with or without H_2_O_2_ for 8 h, and then, an FCM assay was performed to analyse the apoptosis rate of HTR-8/SVneo cells according to the manufacturer’s instructions for the apoptosis detection kit. (b) Decidual macrophages from healthy pregnant women and RSM patients were incubated with apoptotic HTR-8/SVneo cells stained with the CMRA probe for 120 min, and then, a FCM assay was performed to analyse the efferocytosis rate of decidual macrophages stained with CD14-FITC and CD45-APC. The efficiency of the phagocytosis was calculated as follows: (CMRA^+^CD45^+^CD14^+^ macrophages/CD45^+^CD14^+^ macrophages) ×100%. (c) The statistical data were analysed by two-sided paired Student’s t tests. **p < 0.01.

To evaluate the scavenging activity of decidual macrophages on apoptotic trophoblasts, H_2_O_2_-induced apoptotic HTR-8/SVneo trophoblasts were stained with a CMRA probe before they were incubated with decidual macrophages. Efferocytosis was investigated by FCM quantification after decidual macrophages were labelled with CD14-FITC and CD45-APC fluorochrome-conjugated antibodies. CD14^+^CD45^+^CMRA^+^ cells are the population of decidual macrophages that engulf apoptotic trophoblasts. As shown, the efferocytosis activity of decidual macrophages was reduced in RSM patients compared with that in healthy pregnant women (Figure 6).

### Phagocytosis is impaired in decidual macrophages upon demethylation treatment

**Figure 7.**
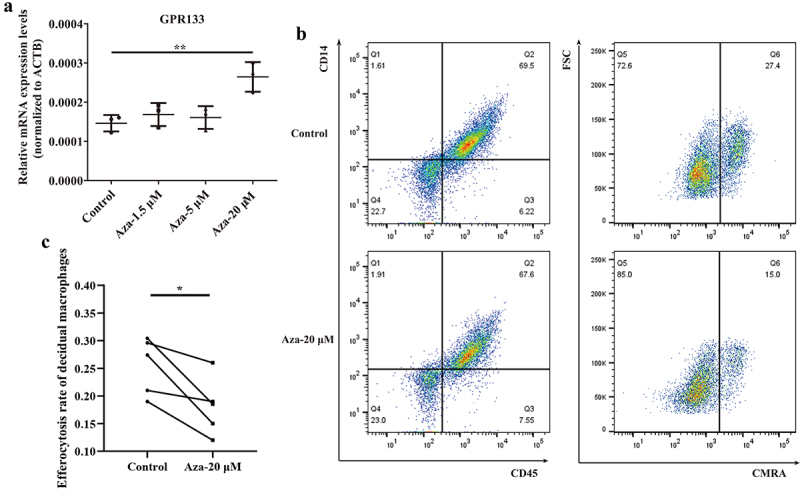
Upregulation of *GPR133* by demethylation in decidual macrophages decreased the percentage of apoptotic HTR-8/SVneo cells that underwent phagocytosis. (a) Decidual macrophages from healthy pregnant women were treated with 5-aza-dc for 72 h; thereafter, the expression of *GPR133* was measured by qRT – PCR. The data are presented as the means ± SDs and were analysed by two-sided paired Student’s t tests. **p < 0.01. (b) Decidual macrophages from healthy pregnant women were subjected to continuous demethylation treatment with 5-aza-dC for 72 h and incubated with apoptotic HTR-8/SVneo cells stained with the CMRA probe for 120 min. A FCM assay was performed to analyse the efferocytosis rate. The efficiency of the phagocytosis was calculated as follows: (CMRA^+^CD45^+^CD14^+^ macrophages/CD45^+^CD14^+^ macrophages) ×100%. (c) The statistical data were analysed by two-sided paired Student’s t tests. *p < 0.05.

To explore the influence of *GPR133* expression on phagocytosis, we performed a demethylation test in normal control decidual macrophages, which exhibited increased methylation (Figure 4) and decreased expression of the *GPR133* gene (Figure 3). In addition, 5-Aza-dC treatment significantly increased *GPR133* mRNA expression by ~ 1.8-fold relative to that in untreated control cells (Figure 7(a)) and markedly impaired phagocytosis (Figures 7(b,c)). These data suggest that promoter methylation is a possible mechanism for the epigenetic silencing of *GPR133* transcription to maintain phagocytosis in decidual macrophages during normal early pregnancy.

### Phagocytosis is enhanced in GPR133-knockdown THP-1 macrophages

**Figure 8.**
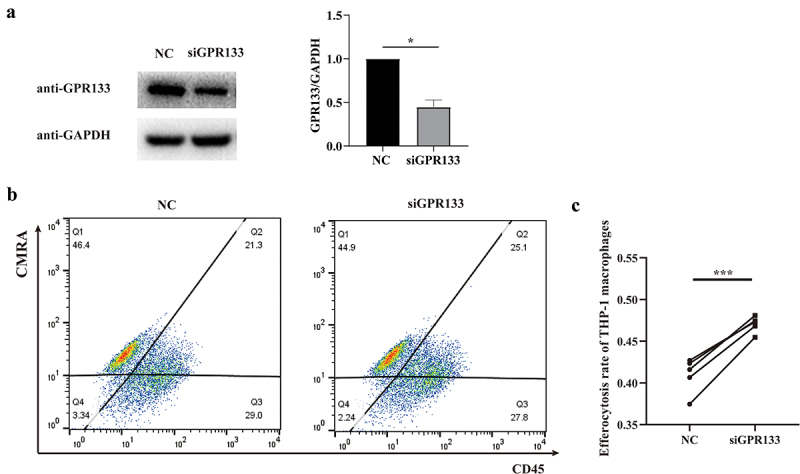
Knockdown of *GPR133* in THP-1-derived macrophages increased the percentage of phagocytosis of apoptosis-induced HTR-8/SVneo cells. (a) THP-1 macrophages were treated with NC or GPR133 siRNA for 48 h. The expression levels of GPR133 were examined via western blotting. Densitometric quantification is shown. The statistical data were analysed by Student’s t test. The data are presented as the means ± SEMs. **p < 0.05. (b) THP-1 macrophages were treated with or without GPR133 siRNA for 48 h and then incubated with apoptotic HTR-8/SVneo cells stained with the CMRA probe for 120 min. A FCM assay was performed to analyse the efferocytosis rate in THP-1 macrophages stained with CD45. The efficiency of the phagocytosis was calculated as follows: (CMRA^+^CD45^+^ macrophages/CD45^+^ macrophages) ×100%. (c) The statistical data were analysed by two-sided paired Student’s t tests. *p < 0.001.

To further validate the influence of GPR133 expression on phagocytosis, *GPR133* was knocked down by siRNA in THP-1 macrophages. Subsequently, THP-1 macrophages were incubated with CMRA-labelled apoptosis-induced HTR-8/SVneo cells and investigated by FCM quantification after staining with CD45-APC fluorochrome conjugated antibodies. The CD45^+^CMRA^+^ cells were the population of THP-1 macrophages that engulfed apoptotic HTR-8/SVneo cells. The phagocytosis of THP-1 macrophages increased after *GPR133* was knocked down (Figure 8).

## Discussion

In the present study, we aimed to identify changes in decidual macrophages from RSM patients compared to those from healthy normal control subjects. *GPR133* was found to be significantly hypomethylated and upregulated in the decidual macrophages from women with RSM. To validate the functional effects of these changes, demethylation and knockdown studies were performed. These findings demonstrate that epigenetic regulation of *GPR133* is a potential aetiological factor for RSM.

DNA methylation is established and maintained mainly by DNA methyltransferases (DNMTs), while removal of methylation relies on ten-eleven translocation enzymes and the thymine DNA glycosylase [13]. DNA methylation occurs throughout the entire reproductive process and inhibits the expression of key genes involved in gametogenesis, embryonic development, trophoblast invasion and maternal-foetal interface formation. The maternal-foetal interface comprises of both the foetal-derived placenta and the maternal-derived decidua, which together maintain maternal-foetal immunotolerance and provide immunologic defences against infection for the foetus. Abnormal DNA methylation can destroy complex immune mechanisms and may potentially cause pregnancy loss [9,14,15]. Du et al. performed a genome-wide DNA methylation analysis of the placental villi from RSM patients and found many significantly differentially methylated regions near dysregulated genes that were enriched in the immune response pathway [9]. DNA methylation analysis of the decidua highlights the role of hypomethylation of *CREB5* in the pathogenesis of RSM through regulation of the immune response [9,15]. Precise analysis of DNA methylation status in specific cell populations is required to better understand the contribution of aberrant epigenetic regulation in response to RSM. It has been fully elucidated that decidual macrophages contribute the second largest decidual leukocyte population at the maternal-foetal interface in the first trimester, next to natural killer cells [4]. Emerging evidence has indicated an association between the disturbance of decidual macrophages and the occurrence of RSM [2]. Our current study sought to integrate DNA methylation data with transcriptome profiles to explore key epigenetic features in decidual macrophages. We observed reduced methylation of *GPR133* in the decidual macrophages of RSM patients, highlighting that *GPR133* hypomethylation in decidual macrophages may play a critical role in the pathogenesis of RSM. Although studies on DNA methylation in decidual macrophages of RSM are lacking, Schuster et al.’s study in preterm placenta showed that abnormal DNA methylation was enriched in the pathway of Fcy receptor-mediated phagocytosis in macrophages [16], suggesting that abnormal DNA methylation in macrophages may contribute to adverse pregnancy outcomes.

During pregnancy, apoptosis is prevalent and critical for the tissue remodelling of maternal decidua and the invasion of the developing embryo [17]. The clearance of apoptotic cells (a process termed efferocytosis) is an active event that prevents the release of self-antigens or cytotoxic contents and is related to the local tolerance at the maternal-foetal interface [18]. Decidual macrophages are the main operators of efferocytosis at the maternal-foetal interface [19]. Several researches have suggested that efferocytosis has anti-inflammatory effects, promoting the M2 polarization of decidual macrophages [20,21]. Therefore, the effective clearance of apoptotic cells by decidual macrophages represents an essential process for alleviating inflammatory environments at the maternal-foetal interface. Defects of apoptotic corpse elimination are closely associated with some undesirable pregnant complications [21]. Consistently, our findings support the point that the efferocytosis of decidual macrophages was reduced in RSM patients compared with healthy pregnant women.

GPR133, also known as *ADGRD1*, is an adhesion G protein – coupled receptor [22]. It was revealed that GPR133 couples with Gαs activating adenylyl cyclase and leading to accumulation of cAMP [23]. It was demonstrated that elevations in intracellular cAMP are associated with suppression of phagocytic activity of macrophages [24,25]. Consistently, our findings support the point that increased levels of *GPR133* expression are associated with inhibition of macrophage phagocytosis. Mechanistically, once cAMP is generated in macrophages, it can activate downstream signalling cascades by binding to the effector proteins such as Ser/Thr phosphorylating enzyme named protein kinase A (PKA) or guanine-nucleotide exchange protein directly activated by cAMP. It seemed that only PKA activation resulted in the suppression of phagocytosis [26]. The cAMP-dependent PKA exists in two major isoforms, including types RI and RII [27]. PKA isoform RI activation appeared to be more important than isoform RII in mediating the inhibition of phagocytosis in phorbol-12-myristate-13-acetate-differentiated THP-1 macrophages [26]. Therefore, it is reasonable to hypothesize that the GPR133-cAMP-PKA isoform RI pathway is involved in the regulation of phagocytosis in decidual macrophages.

A significant correlation between *GPR133* expression and DNA methylation was observed in oral squamous cell carcinoma [28]. In this study, we found that a new differentially methylated region near the transcription start site of *GPR133* was hypomethylated in the decidual macrophages of RSM patients. Hypomethylation in the regulatory region opens the chromatin region, exposes the DNA sequence to transcription factors, recruits cofactors, and eventually affects the gene transcription [9]. It is widely accepted that DNA methylation profiles are dynamic and prone to modifications in response to environmental changes, nutritional factors, lifestyle factors and other factors. Environmental cues, such as increased levels of inflammatory cytokines, can modify DNA methylation patterns [29]. Nutrients have been shown to modify DNA methylation by acting as coenzymes, inducing the formation of methyl donors, or modifying DNMT enzymatic activity [30]. There is evidence indicating that maternal adverse lifestyles may affect the intrauterine environment by altering DNA methylation, resulting in adverse pregnancy outcomes such as abortion [31,32]. In addition, the DNA methylation pattern is also associated with ageing [33]. Physiologic ageing increases DNA methylation in oocytes [34], providing a better understanding of the mechanisms underlying the decrease in oocyte quality and reproductive potential of aged females.

This study has several limitations. The first may be that the results lacked *in vivo* experimental validation, which should be addressed in further studies. Second, it was difficult to investigate the effect of *GPR133* knockdown on phagocytosis in primary decidual macrophages from RSM patients due to the small amount of fresh tissue remaining after clinical diagnosis. Third, RSM is a multifactorial disease. Integrated studies of multiple cells in addition to decidual macrophages at the maternal-foetal interface need to be performed.

In summary, we propose a model in which hypomethylation at the *GPR133* promoter induces *GPR133* expression. This increase in expression results in decreased efferocytosis of decidual macrophages, leading to RSM. Importantly, these findings suggest that the methylation level of *GPR133* may be a promising target for intervention in RSM and a promising biomarker for early diagnosis of RSM. We believe that early identification of new prognostic markers would be of great help to obstetricians for the early detection and management of RSM patients.

## Supplementary Material

-)Supplementary tables.docx

## Data Availability

The datasets used and/or analysed during the current study are available from the corresponding author on reasonable request.
