# Supplementary material for: Upregulation of GPR133 expression impaired the phagocytosis of macrophages in recurrent spontaneous miscarriage
Source: Epigenetics. 2024 Apr 2;19(1):2337087. doi: 10.1080/15592294.2024.2337087 (PMC10989699; doi:10.1080/15592294.2024.2337087)
Supplement: -)Supplementary tables.docx [file KEPI_A_2337087_SM2865.docx]

**Supplementary tables**

**Supplementary Table S1. Subject demographics**

| **Characteristics** | **Normal^a^**  **(N=26)** | **RSM^b^**  **(N=21)** | **P value^c^** |
| --- | --- | --- | --- |
| Age (years) | 30.04±1.17 | 32.14±0.68 | 0.1278 |
| Gestational age (days) | 50.41±1.53 | 59.68±2.21 | 0.001 |
| First trimester loss (times) | 0±0 | 2.62±0.18 | <0.001 |

^a^ Normal subjects were women with clinically healthy pregnancies that had been terminated voluntarily for nonmedical reasons. Data are presented as the means ± SEMs.

^b^ RSM subjects were abortion patients who had experienced more than two unexplained and consecutive spontaneous abortions. Data are presented as the means ± SEMs.

^c^ P-value was calculated by two-tailed unpaired student’s t-test for normally distributed data (age and gestational age) or two-tailed Mann Whitney test for non-normally distributed data (first trimester loss).

**Supplementary Table S2. Primer sequence**

| **Name** | **Sequence** | **Roles** |
| --- | --- | --- |
| RHBDF1-F | GTTTTTGGGATTTTTAGTTGTTG | BSP |
| RHBDF1-R | AACTTTTCTTCCTAAAATTTCCC | BSP |
| EPAS1- F | TAATATTGGATGTTTTTGGTAGGTT | BSP |
| EPAS1-R | AAAATTAAAACCCTACCTACTTCTAC | BSP |
| SLC44A4-F | GTTGTTAGAGATAGTAGTAGGAAGAT | BSP |
| SLC44A4-R | CTACTTAAAAACCAATTAAAATCC | BSP |
| TRP4-F | TTTTTTGGATAAAGTTAAGAGTTTTTA | BSP |
| TRP4-R | CTCCACCTCCAAACAATATAATAC | BSP |
| GPR133-F | GGGGAGATTTTTATTATTGAAGTG | BSP |
| GPR133-R | AACCTAAATCCTCCATCTCAAAC | BSP |
| RHBDF1-F | CGAGACAGCCCACATCTCTTC | qPCR |
| RHBDF1-R | GCTCACTCCAAACCAGTCGG | qPCR |
| EPAS1- F | ATAAGTTCACCCAAAACCCCAT | qPCR |
| EPAS1-R | GGCAGCAGGTAGGACTCAAAT | qPCR |
| SLC44A4-F | CTGGCTACATCGGGGCAAC | qPCR |
| SLC44A4-R | CACGAGGAGTTCACAAGGTG | qPCR |
| TRP4-F | GGACTTCAGGACTACATCCA | qPCR |
| TRP4-R | ACGCAGAGAACTGAAGATGT | qPCR |
| GPR133-F | GAAGGGAAGGTCAACAAAGG | qPCR |
| GPR133-R | CACTGCTCTGGCTTGCTGAT | qPCR |
| DNMT1-F | CAGGCAAACCACCATCACATC | qPCR |
| DNMT1-R | CTTCTCATCCTGGTCTTTGTCTT | qPCR |
| β-actin-F | AGCCTCGCCTTTGCCGAT | qPCR |
| β-actin-R | CTTCTGACCCATGCCCACC | qPCR |
| GAPDH-F | GAGTCAACGGATTTGGTCGT | qPCR |
| GAPDH-R | CATGGGTGGAATCATATTGGA | qPCR |

**Supplementary Table S3. Expression levels of genes related to DNA methylation regulation**

| **Genes** | **Description** | **Fold change** | **P value** | **Normalized signal (log2)** | | | | | |
| --- | --- | --- | --- | --- | --- | --- | --- | --- | --- |
|  |  |  |  | **NOR-1** | **NOR-2** | **NOR-3** | **RSM-1** | **RSM-2** | **RSM-3** |
| DNMT1 | DNA (cytosine-5-)-methyltransferase 1 | 0.33097 | 0.00017 | 9.8639 | 9.8051 | 10.0869 | 8.2664 | 8.2262 | 8.4801 |
| MBD4 | methyl-CpG binding domain protein 4 | 0.44082 | 0.00185 | 9.2486 | 9.3892 | 9.3681 | 8.3021 | 7.9788 | 8.1657 |
| HDAC8 | histone deacetylase 8 | 0.46883 | 0.00362 | 7.3117 | 7.1192 | 7.2355 | 5.9069 | 6.2939 | 6.1669 |
